# Knowledge on Parental Hesitancy toward COVID-19 Vaccination of Children 5–11 Years Old

**DOI:** 10.3390/vaccines11030587

**Published:** 2023-03-03

**Authors:** Susanna Esposito, Cristiano Rosafio, Simonetta Partesotti, Michele Fiore, Francesco Antodaro, Andrea Bergomi, Cosimo Neglia, Alberto Argentiero, Nicola Principi, Stefano Zona

**Affiliations:** 1 Pediatric Clinic, Pietro Barilla Children’s Hospital, Department of Medicine and Surgery, University of Parma, 43126 Parma, Italy; 2 Primary Care Pediatricians, Local Health Agency of Modena, 41121 Modena, Italy; 3 Primary Health Care Department, Local Health Agency of Modena, 41121 Modena, Italy; 4 Primary Care Pediatricians, Local Health Agency of Genoa (ASL3), 16129 Genoa, Italy; 5 Università degli Studi di Milano, 20122 Milan, Italy; 6 Health Direction, Local Health Agency of Modena, 41121 Modena, Italy

**Keywords:** children, COVID-19, COVID-19 vaccines, pediatric infectious diseases, vaccine hesitancy

## Abstract

Although vaccines are the safest and the most effective measure to prevent disease, disability, and death from various pediatric infectious diseases, parental vaccine hesitancy is a common and increasing phenomenon worldwide. To contribute to improving our knowledge on parental willingness and hesitancy toward COVID-19 vaccine administration in children aged 5–11 years, an anonymous online questionnaire was disseminated in Italy after the COVID-19 vaccine’s authorization for this age group. An online survey was conducted using the Crowd Signal platform from 15 December 2021 to 15 January 2022 in Italy among parents of children 5–11 years old. A total of 3433 questionnaires were analyzed. Overall, a “Favorable” position was observed in 1459 (42.5%) parents, a “Doubtful” one in 1223 (35.6%) and a “Hesitant/Reluctant” one in 751 (21.9%). The univariate multinomial logistic regression analysis and the multivariate multinomial logistic regression analysis showed that the Hesitant/Reluctant parents were younger than 40 years of age, mostly female, with a secondary or middle school degree, an annual income below EUR 28,000, more than one child in the age range from 5 to 11 years, an underestimated consideration of the severity of COVID-19’s effects, and concern regarding the COVID-19 vaccines in general. These results show that in Italy, most parents of children aged 5 to 11 were doubtful or hesitant/reluctant to vaccinate their children against the COVID-19 virus. Poor trust in health institutions as well as poor consideration of the epidemiological and clinical relevance of COVID-19 in children seem to have played the biggest roles in forming these attitudes. Moreover, the negative attitude of several parents who previously agreed to immunize their children against other childhood illnesses according to the official national pediatric immunization schedule clearly indicates that only the COVID-19 vaccine was put in doubt or rejected. All these findings lead us to conclude that to improve COVID-19 vaccination coverage in children aged 5 to 11, health authorities should increase parental education on the true clinical relevance of COVID-19 and on the importance of its prevention to hinder the evolution of the pandemic in pediatric subjects and the emergence of new variants, and its relative weight in influencing the efficacy of vaccines.

## 1. Background

Although vaccines are the safest and the most effective measure to prevent disease, disability, and death from various pediatric infectious diseases [1], parental vaccine hesitancy, defined by the World Health Organization as “delay in acceptance or refusal of vaccines despite availability of the vaccine services”, is a common and increasing phenomenon worldwide [2,3]. Studies have shown that up to 50% of parents have concerns about immunizing their children with the officially recommended pediatric vaccines, even in countries with the most advanced national health systems, such as the USA [4], Canada [5], Australia [6] and several European countries [7,8]. Moreover, it has been reported that the rate of parental hesitancy has increased. In the USA, parental concern about child immunization was declared to be 20% in the year 2000 [9], and by 2009, this figure had risen to 50% [10].

Parental vaccine hesitancy is one of the most important reasons that administration of some pediatric vaccines remains far from optimal, and outbreaks of certain vaccine-preventable diseases can occur when coverage does not reach the necessary level needed to develop herd immunity [11,12,13]. The very recent outbreaks of measles in several industrialized countries are the best example of this concern [14].

Generally, parental hesitancy does not concern all infectious disease vaccines that are recommended by health authorities for child protection. Only 1–2% of parents totally exclude childhood immunization [15]. More commonly, one or more vaccines which are refused or debated with greater prevalence are those that are new or totally unknown, have relatively undefined or debated safety and efficacy outcomes, or that require repeated administrations and cannot be given on a delayed schedule [9,15,16].

To counter the COVID-19 pandemic, active immunization with specific vaccines is considered essential; several vaccines obtained with different methods were developed, and some of them were evaluated in clinical trials for use in humans [17,18]. Initially, trials were carried out in adults, given that COVID-19 showed greater prevalence the older the patients were, and showed more serious symptoms and outcomes in older people. Later, pediatric formulations were prepared, and most national health authorities, including the U.S. Food and Drug Administration (FDA) and the European Medicines Agency (EMA), authorized their use when clinical trials certified that they could be considered safe and sufficiently effective in children of different ages [19,20]. Evidence that COVID-19 could be a severe disease [21,22], that it could be associated with serious or very unpleasant long-term consequences, such as multisystem inflammatory syndrome in children (MIS-C) [23] and long-COVID [24,25], and that reducing pediatric COVID-19 cases would reduce virus circulation and risk of disease in high-risk populations [26,27] were the main reasons that health authorities suggested supporting the administration of COVID-19 vaccines in children.

Unfortunately, parents’ willingness to vaccinate their children against COVID-19 has always turned out to be rather low, even lower than their willingness to be vaccinated themselves. In Italy, the intention to accept a COVID-19 vaccine of parents of children <18 years of age was 60.4%, compared to values of 64.5% and 84.1% found in the general population [28,29,30]. Detailed knowledge of parental willingness and hesitancy toward pediatric COVID-19 immunization is essential to understand the reasons for non-vaccination and to prepare adequate measures to improve vaccination coverage. Most of the currently available data regarding vaccination come from the first age group for which COVID-19 vaccines were authorized, that is, for older children aged 12 to 17. Moreover, some of the data regarding children aged 5 to 11 years were collected before pediatric COVID-19 vaccine was authorized for use, and indicate only the parental intention, independent of the suggestions of most health authorities [31,32,33,34,35]. To contribute to improving our knowledge on parental willingness and hesitancy towards COVID-19 vaccine administration in children aged 5 to 11 years, an anonymous online survey was conducted in Italy after the COVID-19 vaccine’s authorization for this age group.

## 2. Methods

The same online questionnaire (Supplementary Material S1), developed by pediatricians and infectious diseases specialists (SZ, SP and AB) of the Local Health Agency of Modena, Italy, previously validated and used to evaluate determinants of vaccine parental hesitancy towards the COVID-19 immunization of adolescents was utilized to perform this survey. The methods for distributing and collecting the completed questionnaires were consistent [30]. The online survey was carried out from 15 December 2021 to 15 January 2022, starting a few weeks after Italy’s medicines agency AIFA approved the use of the BNT162b2 vaccine (Comirnaty, Pfizer) in children aged 5 to 11 years [36].

A total of 3500 parents of children 5–11 (i.e., 10% of the Italian parents of children in this age group) received the questionnaire. Answers (67) that had completion times below 4 min were discarded. Data collected in this study were analyzed using the same methods as in the previous study [30].

The demographic differences between the groups were evaluated using parametric methods (ANOVA) for continuous variables with normal distribution, and non-parametric methods (Kruskal–Wallis test) for continuous variables with non-normal distribution. Categorical variables were analyzed using the X2 test or Fisher’s exact test. The scores on the statements placed in the various sections of the questionnaire and relating to the information scores were described with box plots; differences in medians were evaluated using the Kruskal–Wallis test. For questions with categorical answers, the X2 test or Fisher’s exact test was used. Finally, a univariate and subsequently multivariate model of multinomial logistic regression was developed, chosen from different significant variables of univariate multinomial logistic regression. Statistical significance was considered for *p*-values < 0.05. All statistical analyses were performed with the STATA 12.1 software (Stata-Corp, College Station, TX, USA). Being a descriptive study, a formal calculation of the sample size was not performed.

## 3. Results

Among 3433 evaluable questionnaires, 2869 were completed by females (83.6%), 548 were completed by males (16.0%), and 16 questionnaires did not indicate gender (0.5%). Table 1 summarizes the demographic characteristics of those who answered the questionnaire. The declared average age was 41.3 years (standard deviation [SD]: 5.3). Most of the questionnaires came from Northern Italy; 1533 were from Emilia-Romagna (46.5%) and 430 were from Lombardy (13.0%). Most people answered that they were in a position of permanent employment (2138, 62.3%) or were self-employed (626, 18.2%). Unemployment was more frequent in females (10.1% vs. 0.7%). Regarding education level, 1172 (34.1%) were high-school graduates and 1108 (32.3%) reported having a Master’s degree. The number of children reported was one child for 1115 questionnaires (32.5%), two children for 1887 (55.0%) and three or more children for 431 (12.5%). Overall, 2485 (72.4%) answered that they had only one child 5–11 years old, whereas 948 (27.6%) answered that they were the parent of more than one child 5–11 years old. Regarding reported family income, the majority reported an income >EUR 28,000 /year. Italians made up the vast majority of those who answered the questionnaires (3381, 98,5%); 9 (0.3%) did not report ethnicity, 15 (0.4%) reported coming from Western Europe, 14 (0.4%) from Eastern Europe, 3 (0.1%) from North Africa, 4 (0.1%) from Asia and 7 (0.2%) from the Americas.

Using a cluster analysis based on the stated agreement level for use of the anti-COVID vaccination in adolescents, three groups were created: “Favorable” 1459 (42.5%), “Doubtful” 1223 (35.6%) and “Hesitant/Reluctant” 751, (21.9%). The three groups reflected different age groups, with a higher prevalence of younger parents in the Hesitant/Reluctant group. In terms of gender, women were more prevalent in the Doubtful group; for work conditions, self-employed and non-responsive people were more present in the Hesitant/Reluctant group; and for declared annual income, the lowest-income people were more prevalent in the Hesitant/Reluctant group.

**Figure 1:**
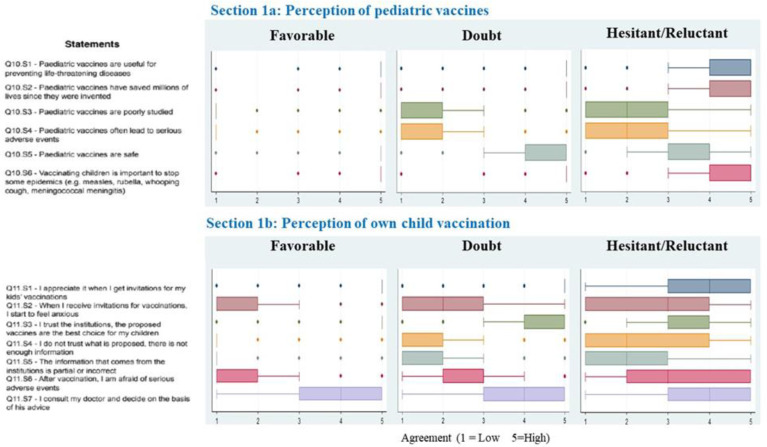
Boxplot of concordance scores of confidence in vaccines and vaccinations. All *p*-values < 0.05 after Bonferroni adjustment.

Figure 1 shows the concordance scores, expressed via boxplot (median and interquartile range), to the statements proposed in Q10. Overall, Favorable and Doubtful parents declared a higher level of confidence in the safety and efficacy of pediatric vaccines than Hesitant/Reluctant parents; this was investigated through statements Q10.S1 and Q10.S5 (all *p*-values < 0.001).

Similar results were reported for the statements included in Q11, which investigated confidence in health institutions in relation to the personal experience of individuals’ own children’s vaccination through statements Q11.S3 and Q11.S3 (all *p*-values < 0.001).

Regarding Question 12, “Did all of your children have pediatric vaccinations according to the recommended schedule?”, 3252 (95.2%) answered “Yes”; 94 (2.8%) answered “No, but due to postponements for health reasons”; 35 (1.02%) answered “No, because I did not have sufficient reassurance”; and 32 (0.94%) did not answer. The lowest frequency of “Yes” answers was found in the Hesitant group (87.7%), against 98.0% and 96.3% in the Favorable and Doubtful groups. Parents who declared “No, because I did not have sufficient reassurance” were more frequent in the Hesitant/Reluctant group; they were 25 (3.4%) versus 1 in the Favorable group (0.1%) and 9 in the Doubtful group (0.7%). Those who answered “No, but due to postponements for health reasons” were 1 (0.1%) in the Favorable, 32 (2.6%) in the Doubtful and 37 (5.0%) in the Hesitant/Reluctant group. Differences in the frequency of “Yes” answers were calculated with the Chi-square test, and were statistically significant lower in the Hesitant/Reluctant group (*p* < 0.001).

**Figure 2:**
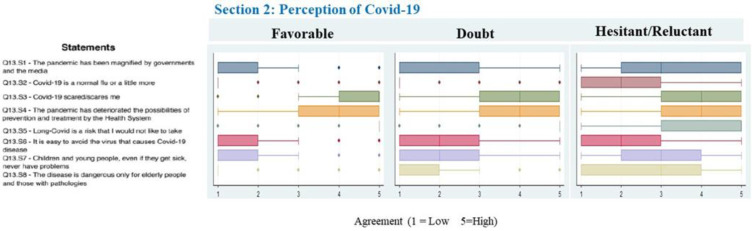
Boxplot of concordance scores on perception of COVID-19. All *p*-values < 0.05 after Bonferroni adjustment.

Figure 2 shows the scores of the statements on the risk perception of COVID-19. Risk perception of COVID-19 differed between groups; Hesitant/Reluctant parents reported a higher level of complacency in themselves and their children than the Favorable and Doubtful groups. In particular, the median score for the statement Q13.S7 was 1 (IQR 1; 2) for the Favorable group, 2 (IQR 1; 3) for the Doubtful group and 3 (IQR 2; 4) for the Hesitant/Reluctant group (Kruskal–Wallis test *p* < 0.001). The perceived ease of avoiding COVID-19 was significantly lower in the Favorable group (Median score: 1, IQR: 1; 1) (Kruskal–Wallis test *p* < 0.001).

Overall, 2195 (63.9%) reported that they or a house mate had had a positive swab; only 18 (0.5%) did not answer, 1581 (46.1%) reported that they or a loved one had symptomatic COVID-19, and 1838 (53.5%) did not have any symptoms. No differences among the three groups were found regarding symptomatic disease. A “Yes” answer to the question of if they or a household member had a positive swab showed a statistically significant difference in the Favorable group, at 62.1%, and in the Hesitant/Reluctant group, at 67.0% (*p* < 0.01).

Regarding the results of Question 16, “Has a family member or loved one died due to COVID-19?”, 642 (18.7%) answered affirmatively, and 20 (0.60%) did not answer. Answers of Favorable and Doubtful parents did not show significant differences.

Doubtful and Hesitant/Reluctant parents were less well informed than Favorable parents, considering median scores to the statement Q17.S1 (“Authorized anti-COVID vaccines are still experimental”), which resulted in 1 (IQR: 1; 1), 2 (IQR: 1; 3) and 4 (IQR: 3; 5) for Favorable, Doubtful and Hesitant/Reluctant groups, respectively, with *p* < 0.001. To the statement Q17.S7 (“Vaccines are the best way to avoid deaths and hospitalizations”), median scores were 5 (IQR: 5; 5), 5 (IQR: 4; 5) and 4 (IQR: 3; 5) for Favorable, Doubtful and Hesitant/Reluctant parents, respectively (*p* < 0.001). Regarding information sources, Favorable and Doubtful parents were more likely to consider general practitioners as authoritative (Q18.S1); median scores were 5 (IQR: 3; 5), 4 (IQR: 3; 5) and 4 (IQR: 2; 5) for Favorable, Doubtful and Hesitant/Reluctant parents, respectively, with *p* < 0.001. Similar findings were observed in doctors working for the National Health System (Q18.S2): 5 (IQR: 3; 5), 4 (IQR: 2; 5) and 3 (IQR: 0; 4) for Favorable, Doubtful and Hesitant/Reluctant, respectively, with *p* < 0.001.

**Figure 3:**
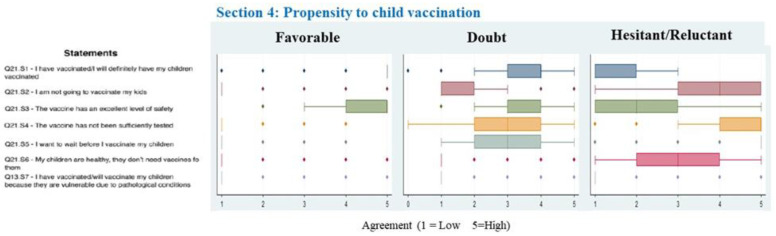
Boxplot of concordance scores on risk perception, safety and propensity for anti-COVID vaccination for adolescents. All *p*-values < 0.05 after Bonferroni adjustment.

The comparison analysis of risk perception, safety and propensity for anti-COVID vaccination in children is described in Figure 3. Regarding individual adult vaccination hesitancy, Favorable and Doubtful parents received or booked significantly more frequent anti-COVID vaccinations: 1457 (99.9%) and 1206 (98.6%) vs. 602 (80.2%), and also recommended the anti-COVID vaccination (1458 (99.9%) and 1174 (96.0%) vs. 472 (62.9%)) more frequently than the Hesitant/Reluctant parents (*p* < 0.001 for all the comparisons).

**Figure 4:**
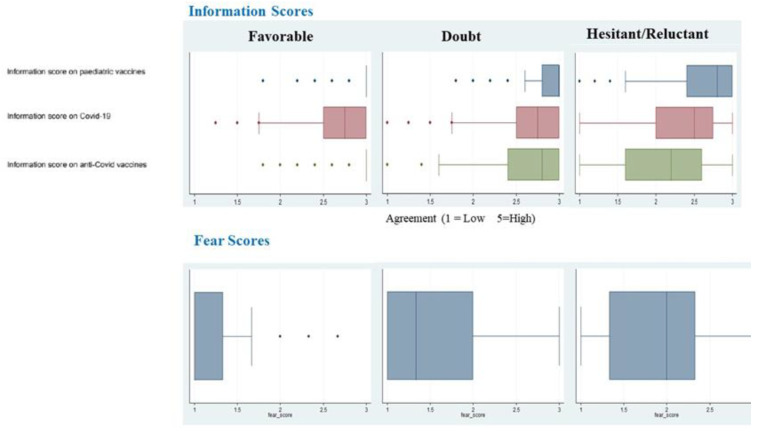
Boxplot of the information scores and the fear score. All *p*-values < 0.05 after Bonferroni adjustment.

Figure 4 shows the information scores and fear scores. Hesitant/Reluctant parents showed lower median scores than Doubtful and Favorable parents with regard to pediatric vaccines: 2.2 (IQR 1.6; 2.6), 2.8 (IQR: 2.4; 3.0) and 3 (IQR: 3.0; 3.0), respectively, with *p* < 0.001. For fear scores on COVID-19, the scores were 2 (IQR 1.3; 2.3) for Hesitant/Reluctant, 1.3 (IQR: 1; 2) for Doubtful and 1 (IQR: 1.0; 1.3) for Favorable, with *p* < 0.001.

The univariate multinomial logistic regression analysis is shown in Table 2. An age lower than 40 years was significantly associated with being Doubtful (*p* < 0.01) and Hesitant/Reluctant (*p* < 0.001). The female gender resulted in being associated with Doubtful and Hesitant/Reluctant (*p* < 0.001). People declaring an education higher than high school were less frequently Doubtful and Hesitant/Reluctant (*p* < 0.01 and *p* < 0.001, respectively), while Hesitancy and Doubtfulness were associated with “no answer” (*p* < 0.001). A declared annual income less than 28,000 Euro (*p* = 0.001) and “no answer” (*p* < 0.001) were associated with Hesitant/Reluctant and Doubtful; “no answer” was associated with being Doubtful and Hesitant/Reluctant (RR = 1.43 and RR = 2.60, *p* < 0.001). The number of children 5–11 years higher than 1 was positively associated with being Hesitant/Reluctant (RR = 1.29, *p* < 0.01). The parent’s area of residence being in the south and in the islands was less likely to be associated with being Hesitant/Reluctant (RR = 0.65, *p* < 0.05).

Table 3 summarizes the findings of multivariate multinomial logistic regression analysis. After multivariate multinomial logistic regression analysis, Doubtful parents were negatively associated with age >40 years (both for 41–50 years old and >50 years old) (*p* < 0.001), male gender (*p* < 0.001), unemployment/unpaid domestic work (*p* < 0.05), information score on pediatric vaccines (*p* < 0.001), and information score on COVID-19 (*p* < 0.001); they were positively associated with fear score (*p* < 0.001). Hesitant/Reluctant parents were negatively associated with age >40 years (*p* < 0.001 both for 41–50 years old and >50 years old), with unemployment/unpaid domestic work (*p* < 0.05), with information score on pediatric vaccines (*p* < 0.001) and on COVID-19 (*p* < 0.001); they were positively associated with retired/no answer (*p* < 0.05) and fear score (*p* < 0.001).

## 4. Discussion

This survey was carried out a few weeks after AIFA had authorized the use of BNT162b2 vaccine in 5–11 year-old children [36]. In this period of time, no official recommendations for its use by the Italian Ministry of Health had been issued, and only a few scientific pediatric societies, including the Italian Society of Pediatrics (SIP), had generically expressed their belief that all children, at least those at greater risk of severe COVID-19 symptoms, should be immunized [37]. This means that despite the limitations of a questionnaire, the results of this study express the real thoughts of parents with children aged 5–11 years about the immunization of their sons and daughters against COVID-19, without major external influences. Consequently, they can elucidate the true barriers to the administration of the COVID-19 vaccine in children aged 5–11, and provide the health authorities with a useful resource to overcome parental hesitancy. The study shows that in Italy, most parents of 5–11 year-old children were doubtful or hesitant/reluctant to vaccinate their children. 

Poor trust in health institutions as well as poor consideration of the epidemiological and clinical relevance of COVID-19 in children seem to have played a major role in forming this attitude. Parents that were doubtful or hesitant/reluctant to accept the COVID-19 vaccine believed that health authorities placed too much importance on this disease. Moreover, they tended to minimize clinical relevance of COVID-19 in children, not highlighting the importance of prevention and stating potential vaccine-related adverse events instead. The lack of reliable data on vaccine safety and effectiveness in children of this age group was underlined. Interestingly, a negative association between inclusion in the group of Doubtful or Hesitant/Reluctant parents and parent age, unemployment/unpaid domestic work, information scores on pediatric vaccines and on COVID-19 was evidenced. These findings underline the role of education and income in encouraging compliance of parents with vaccine recommendations, particularly when, as is the case with COVID-19 vaccines, both the characteristic of the disease and the global impact of the prophylactic measures are initially not precisely defined. Furthermore, results indicate that the negative attitude towards COVID-19 immunization could be documented even among several parents who have previously agreed to immunize their children according to the official national pediatric immunization schedule. Despite the high recommended vaccine coverage, this could be due in part to the fact that these vaccines are mandated by law in Italy [38]; this finding seems to suggest that in most cases, not all vaccines, but only the vaccine against COVID-19 is the object of doubt or rejection, and that hesitancy toward the COVID-19 vaccine is part of general vaccine hesitancy only in very few cases. Most children who have received the recommended pediatric vaccinations and were born in families that are hesitant/reluctant towards the COVID-19 vaccine could be strongly influenced by the fact that usually, recommended vaccines are mandatory by law in Italy. Interestingly, most parents, regardless of their attitude towards vaccination, indicated that they rely on their general practitioner’s advice regarding vaccines. This seems to suggest that bolstering physicians’ knowledge and intervention may solve most of parents’ doubts and improve vaccination coverage. Conveying information about the real safety and efficacy of COVID-19 vaccines through the GP, within clear recommendations from the Ministry of Health and other major competent authorities, may represent the simplest and most effective method to overcome the parent hesitancy. 

The results of this study are not surprising, as quite similar data were reported in studies regarding parental hesitancy toward COVID-19 immunization of 5–11 year-old children before or immediately after official authorization in a different Italian population [39] and other countries [40,41]. On the other hand, these factors have often been identified among the main reasons for parents’ refusal or lack of conviction to vaccinate their children, even in many studies specifically planned to evaluate the willingness of parents to be compliant with the national recommendations on child vaccination [42,43].

Parental hesitancy systematically translates to very low COVID-19 immunization rates. In Italy, as of 2 February 2022, only 38.5% of the population aged 5–11 years had received at least one vaccine dose, whereas 35.3% had been given a 2-dose vaccination series [44]. However, it cannot be excluded that future surveys may demonstrate different results. A significant improvement in vaccination coverage could occur if the health authorities took a firm pro-vaccination stance, and personal physicians made an effort to convince doubtful and hesitant/reluctant parents. On the contrary, a further increase in parental hesitancy levels with further reduction of COVID-19 immunization rates in children aged 5–11 years may be evidenced by of the impact of the Omicron SARS-CoV-2 variants and the position taken by some countries regarding the administration of COVID-19 vaccines in children aged 5–11 years. Studies carried out after the emergence of the Omicron variant in December 2021 and published some months later showed that the effectiveness of the BNT162b2 vaccine in 5–11 year-old children was significantly reduced compared to the effectiveness shown in the studies performed for emergency authorization and carried out when the wild virus or earlier variants, including the Delta variant, were circulating [45,46,47,48,49]. A retrospective study carried out in Italy between 17 January and 13 April 2022 which enrolled 2,965,918 immunized children and 1,441,166 controls showed that the effectiveness of the recommended two doses of the BNT162b2 vaccine against infection was only 29.4% and 27.4%, respectively, in fully vaccinated and partially vaccinated children [50]. Moreover, the duration of protection was very short, as only 21.2% of immunized children were still protected after 43–84 days from immunization. In light of these results, health authorities had different reactions. Some, like those in the USA, decided to recommend a booster shot using a bivalent vaccine targeting the BA.4 and BA.5 Omicron subvariants [51]. Other experts, mainly those of the UK and Sweden, preferred to re-analyze the cost/benefit ratio problem of vaccine use in younger children [52]. It was highlighted that the Omicron variant was generally associated with a very low risk of severe COVID-19 in children [53]; the incidence of MIS-C was declining during the Omicron wave, despite low rates of COVID-19 vaccination [54]; vaccination could reduce but not prevent virus transmission [55] and, consequently, the indirect protection of others in the family, school or community through child immunization could be marginal. Finally, the decision to administer a third dose was criticized because, as was already reported in adults [56], it was deemed that its protective effect could wane in few weeks. The conclusion was that mRNA COVID-19 vaccines could be considered non-essential for healthy children, and recommended only for patients at increased risk of severe COVID-19 with underlying severe comorbidities [57].

## 5. Conclusions

In Italy, most parents of children 5–11 years old were doubtful or hesitant/reluctant to vaccinate their children against COVID-19. It seems likely that both the evidence of a reduced effectiveness of vaccines and the doubts raised by some experts on the importance of child vaccination could negatively influence attitudes of both parents and pediatricians toward vaccinating children in this age group. Many of the already hesitant parents could find confirmation of their doubts and decide to permanently cancel the vaccination administration. Some pediatricians agree with the experts’ arguments against vaccination, and have negatively influence parents’ choices, especially those parents in the hesitant group. On the other hand, the evidence highlighted by the Italian Ministry of Health states that children of 5–11 years old can safely receive the vaccine; however, the Ministry of Health does not recommend its administration, and states that it can only increase the perplexities and doubts about the use of the vaccine in the aforementioned subjects. Only further studies evaluating the evolution of the pandemic in pediatric subjects, the emergence of new variants and the relative weight influencing the efficacy of the vaccines can eliminate the distrust of governments, health institutions and experts and favor parent decision about COVID-19 vaccine administration to children aged 5–11 years. Moreover, new and more effective vaccines are needed to increase acceptability of COVID-19 vaccination for parents of children 5–11 years old. In the meantime, primary care pediatricians remain the most important point of reference for parents. Generally, these physicians play a key role in educating and advising parents about a vaccine, enabling them to make informed decisions regarding immunization of their children. Primary care pediatricians know which children can be at risk of severe COVID-19 and should make every effort to convince parents of the need to immunize these children, regardless of the importance of prevention in otherwise healthy subjects.

## Figures and Tables

**Table 1:** Demographic characteristics of parents of children 5–11 years old who answered the questionnaire.

Data	Total	Male N = 548	Female N = 2869	*p*
Age, years	41.3 ± 5.3	43.6 ± 6.0	40.8 ± 5.0	<0.001
Group of age, years				<0.001
<40	1573 (45.8%)	181 (33.0%)	1381 (48.1%)	
41–50	1656 (48.24%)	291 (53.1%)	1361 (47.4%)	
>50	204 (5.9%)	76 (13.9%)	121 (4.4%)	
Education				<0.01
Lower secondary school	136 (4.0%)	28 (5.1%)	108 (3.8%)	
High school	1172 (34%)	203 (37.0%)	966 (33.7%)	
BA	508 (14.8%)	64 (11.7%)	442 (15.4%)	
MA	1108 (32.35)	154 (28.1%)	947 (33.0%)	
PhD	419 (12.2%)	87 (15.9%)	330 (11.5%)	
No answer	90 (2.62%)	12 (2.2%)	76 (2.65%)	
Work condition				<0.001
Permanent employment	2138 (62.3%)	379 (69.2%)	1704 (61.1%)	
Temporary employment	253 (7.4%)	21 (3.8%)	232 (8.1%)	
Self-employed	626 (18.2%)	135 (24.6%)	487(17.0%)	
Unemployed/unpaid work	293 (8.5%)	4 (0.7%)	289 (10.1%)	
Retired	7 (0.2)	3 (0.5%)	4 (0.1%)	
No answer	116 (3.38%)	6 (1.1%)	103 (3.6%)	
Number of children				0.30
1	1115 (32.5%)	188 (34.3%)	921 (32.1%)	
2	1887 (55.0%)	285 (52.0%)	1594 (55.6%)	
>2	431 (12.6%)	75 (13.7%)	354 (12.3%)	
Number of children 5–11 years old				0.56
1	2485 (72.4%)	2802 (72.6%)	391 (71.3%)	
>1	948 (27.6%)	787 (27.4%)	157 (28.7%)	
Nationality				0.67
Italian	3381 (98.5%)	538 (98.1%)	2829 (98.6%)	
Foreign	43 (1.3%)	98 (1.6%)	34 (1.2%)	
No answer	9 (0.3%)	1 (0.2%)	6 (0.2%)	
Group				<0.001
Favorable	1223 (35.6%)	300 (57.8%)	1159 (40.4%)	
Doubtful	1459 (42.5%)	149 (27.2%)	1072 (37.4%)	
Hesitant/Reluctant	751 (21.9%)	99 (18.1%)	638 (22.2%)	

**Table 2:** Univariate multinomial logistic regression analysis.

Independent Variable	Favorable	Doubtful		Hesitant/Reluctant	
		RR	95% CI	RR	95% CI
Age, years	1 (Ref.)				-
<40		1 (Ref.)		1 (Ref.)	
41–50		0.73	0.62–0.85	0.55	0.45–0.66
>50		0.62	0.45–0.86	0.39	0.25–0.60
Gender	1 (Ref.)				-
Female		1 (Ref.)		1 (Ref.)	
Male		0.54	0.43–0.66	0.60	0.46–0.76
No answer/non-binary		0.81	-	0.95	-
Education	1 (Ref.)				0.83–2.01
Lower secondary school		1.01	0.65–1.54	1.30	
High school		1 (Ref.)		1 (Ref.)	
BA		0.95	0.75–1.20	0.67	0.51–0.89
MA		0.74	0.62–0.90	0.59	0.47–0.73
PhD		0.55	0.42–0.71	0.39	0.28–0.53
No answer		2.07	1.14–3.75	3.24	1.80–5.85
Work condition	1 (Ref.)				
Employed		1 (Ref.)		1 (Ref.)	
Self-employed		0.92	0.76–1.13	1.12	0.89–1.41
Unemployed/unpaid		0.92	0.70–1.21	0.96	0.69–1.32
Retired/no answer		1.63	1.00–2.65	3.89	2.44–6.19
Annual income	1 (Ref.)				1.34–3.00
<15,000		1.36	0.95–1.97	2.01	
15,001–28,000		1.25	1.02–1.53	1.47	1.15–1.88
28,001–55,000		1 (Ref.)		1 (Ref.)	
55,001–75,000		0.80	0.62–1.02	0.69	0.49–0.95
>75,000		0.67	0.50–0.88	0.92	0.67–1.27
No answer		1.43	1.12–1.82	2.60	1.99–3.38
Number of children	1 (Ref.)				-
1		1 (Ref.)		1 (Ref.)	
2		0.86	0.73–1.01	1.10	0.90–1.33
>2		0.72	0.55–0.92	1.12	0.84–1.50
Number of children 5–11 yrs	1 (Ref.)				-
1		1 (Ref.)		1 (Ref.)	
>1		0.95	0.80–1.13	1.29	1.06–1.56
Nationality	1 (Ref.)				-
Italian		1 (Ref.)		1 (Ref.)	
Foreign		0.84	0.77–0.90	1.62	0.78–3.31
No answer		-		-	
Area of residence	1 (Ref.)				
North		1 (Ref.)		1 (Ref.)	
Center		1.01	0.81–1.25	0.82	0.63–1.08
South and Islands		0.86	0.62–1.19	0.65	0.43–1.00
No answer		2.63	1.61–4.30	4.76	2.93–7.74

CI, confidence interval; RR, relative risk.

**Table 3:** Multivariate multinomial logistic regression.

Independent Variable	Favorable	Doubtful		Hesitant/Reluctant	
		RR	95% CI	RR	95% CI
Age	1 (Ref.)				-
<40 years		1 (Ref.)		1 (Ref.)	
41–50		0.70	0.59–0.83	0.57	0.45–0.72
>50		0.63	0.44–0.90	0.32	0.18–0.57
Gender	1 (Ref.)				-
Female		1 (Ref.)		1 (Ref.)	
Male		0.66	0.52–0.83	0.68	0.48–0.95
No answer/non-binary		0.15	-	-	
Work condition	1 (Ref.)				
Employed		1 (Ref.)		1 (Ref.)	
Self-employed		1.01	0.81–1.26	1.15	0.85–1.54
Unemployed/unpaid		0.70	0.52–0.95	0.61	0.40–0.93
Retired/no answer		1.35	0.80–2.33	2.10	1.11–3.96
Inf. score on vaccines (pre-pandemic)	1 (Ref.)	0.25	0.15–0.42	0.12	0.07–0.22
Inf. score on COVID-19	1 (Ref.)	0.40	0.30–0.54	0.05	0.04–0.08
Fear Score	1 (Ref.)	5.25	13.96–432	12.67	9.90–16.20

CI, confidence interval; RR, relative risk.

## Data Availability

All the available data is included in the manuscript.

## Supplementary Materials

The following supporting information can be downloaded at: https://www.mdpi.com/article/10.3390/vaccines11030587/s1. S1: Questionnaire of the survey.
