# Supplementary material for: Knowledge on Parental Hesitancy toward COVID-19 Vaccination of Children 5–11 Years Old
Source: Vaccines (Basel). 2023 Mar 3;11(3):587. doi: 10.3390/vaccines11030587 (PMC10051409; doi:10.3390/vaccines11030587)
Supplement: Supplementary file 1 [file vaccines-11-00587-s001.zip › vaccines-2188691-supplementary.pdf]

Supplementary Material S1. Questionnaire of the survey.

The questionnaire consisted of 5 sections:

- Collection of demographic data (i.e., age, gender, area of residence, educational qualification, occupation, annual income of the family unit, number of children, number of children between 5 and 11 years of age, nationality);
- Acceptability and general vaccine in the pre-pandemic era;
- Perception of the risk of COVID-19 disease;
- Perception of the safety and efficacy of anti-COVID vaccines;
- Propensity for anti-COVID vaccination for oneself and for children.

The 3 clusters (i.e., "Favorable", "Doubtful" and "Hesitant/Reluctant") were defined by using the method of grouping by median (k-medians) with Euclidean distance of the answers to the question Q21:

- I had my children vaccinated/I will definitely have my children vaccinated;
- I have no intention of vaccinating my children;
- The vaccine has an excellent level of safety;
- The vaccine has not been sufficiently tested;
- I want to wait before vaccinating;
- My children are healthy, so they do not need vaccines;
- I have vaccinated/will vaccinate my children because they are vulnerable to pathological conditions.

Information Scores were also constructed based on the agreement of some statements. The Information Score on pediatric vaccines was constructed with the arithmetic mean of the following scores:

- Q10.S1. Pediatric vaccines are useful for preventing life-threatening diseases (3 for values ≥4; 2 for values equal to 3; 1 for values ≤2);
- Q10.S2. Pediatric vaccines have saved millions of lives since they were invented (3 for values ≥4; 2 for values equal to 3; 1 for values ≤2);
- Q10.S3. Pediatric vaccines are poorly studied (1 for values ≥4; 2 for values equal to 3; 3 for values ≤2);
- Q10.S4. Pediatric vaccines often lead to serious adverse events (1 for values ≥4; 2 for values equal to 3; 3 for values ≤2);
- Q10.S6. Vaccinating children is important to stop some epidemics (i.e., measles, rubella, pertussis and meningococcal meningitis) (3 for values ≥4; 2 for values equal to 3; 1 for values ≤2).

The Information Score on COVID-19 was constructed with the arithmetic mean of the following scores:

- Q13.S2. COVID-19 is a normal influenza or little more (1 for values ≥4; 2 for values equal to 3; 3 for values ≤2);
- Q13.S4. The pandemic has deteriorated the possibilities of prevention and treatment by the health system (3 for values ≥4; 2 for values equal to 3; 1 for values ≤2);
- Q13.S7. Children and young people, even if they get sick, never have COVID-19-related problems (1 for values 4; 2 for values equal to 3; 3 for values ≤2);
- Q13.S8. COVID-19 is dangerous only for the elderly and those with pathologies (1 for

Values _4; 2 for values equal to 3; 3 for values _2).
The Information Score on anti-COVID vaccines was constructed with the arithmetic mean of the following scores:

- Q17.S1. Authorized anti-COVID vaccines are still experimental (1 for values ≥4; 2 for values equal to 3; 3 for values ≤2);
- Q17.S2. Many of the dangerous effects on the health of the recipient of the anti-COVID vaccines are not known (1 for values ≥4; 2 for values equal to 3; 3 for values ≤2);
- Q17.S4. Anti-COVID vaccines are useless to contain the spread of the virus (1 for values ≥4; 2 for values equal to 3; 3 for values ≤2);
- Q17.S5. Anti-COVID vaccines will give rise to dangerous variants of the virus (1 for values ≥4; 2 for values equal to 3; 3 for values ≤2);
- Q17.S7. Anti-COVID vaccines are the best way to avoid deaths and hospitalizations (3 for values ≥4; 2 for values equal to 3; 1 for values ≤2);

Similarly, a Fear Score of vaccines was constructed by using the scores on the following statements:

- Q11.S2. When I receive invitations for vaccinations, I start to feel anxious (3 for values
≥4; 2 for values equal to 3; 1 for values ≤2);
- Q11.S3. I trust the institutions; the proposed vaccines are the best choice for my children (1 for values ≥4; 2 for values equal to 3; 3 for values ≤2);
- Q11.S6. After vaccination, I am afraid of serious adverse events (3 for values ≥4; 2 for values equal to 3; 1 for values ≤2).

**For the parent**<br>Did you give your consent to participate in this survey and did your child agree?<br>Yes No<br>If yes, please continue	
**Demographic Data**	
Q1	Age
Q2	Gender
	M<br>F<br>Non-binary<br>I prefer not to answer
Q3	CAP (residence)
Q4	Education

	Lower secondary school diploma<br>High school diploma<br>BA<br>MA<br>PhD<br>I prefer not to answer
Q5	Work condition
	Permanent employee<br>Temporary employee<br>Self-employed<br>Retired<br>Unemployed - Unpaid domestic worker<br>I prefer not to answer
Q6	Annual family income
	<15,000€<br>15,001-28,000€<br>28,001-55,000€<br>55,001-75,000€<br>>75,000€<br>I prefer not to answer
Q7	Number of children
Q8	Number of children between 12 and 17 of age
Q9	Nationality
	Italy<br>Western Europe<br>Eastern Europe<br>North Africa<br>Central Africa<br>Asia, Americas<br>I prefer not to answer)
**Data on pre-pandemic vaccine hesitancy**	
Q10	Think back to recommended or mandatory paediatric vaccinations: how much do you agree with the following statements? Cast a vote from 1 (disagree) to 5 (fully agree)
Q10.S1	Paediatric vaccines are useful for preventing life-threatening diseases
Q10.S2	Paediatric vaccines have saved millions of lives since they were invented

Q10.S3	Paediatric vaccines are poorly studied
Q10.S4	Paediatric vaccines often lead to serious adverse events
Q10.S5	Paediatric vaccines are safe
Q10.S6	Vaccinating children is important to stop some epidemics (e.g. measles, rubella, whooping cough, meningococcal meningitis)
Q11	We ask you to rethink your experience with your children's paediatric vaccination process: how much do you agree with the following statements? Cast a vote from 1 (disagree) to 5 (fully agree)
Q11.S1	I appreciate it when I get invitations for my kids 'vaccinations
Q11.S2	When I receive invitations for vaccinations, I start to feel anxious
Q11.S3	I trust the institutions, the proposed vaccines are the best choice for my children
Q11.S4	I do not trust what is proposed, there is not enough information
Q11.S5	The information that comes from the AUSL or from the institutions is partial or incorrect
Q11.S6	After vaccination, I am afraid of serious adverse events
Q11.S7	I consult my doctor and decide on the basis of his advice
Q12	Did all of your children have paediatric vaccinations according to the recommended schedule?
	No, because I didn't have enough reassurance<br>Yes<br>No but for delays due to health reasons<br>I prefer not to answer
**Data on Covid-19 risk perception**	
Q13	We ask you to rethink your experience throughout the pandemic period, from March 2020 to today: how much do you agree with the following statements? Cast a vote from 1 (disagree) to 5 (fully agree)
Q13.S1	The pandemic has been magnified by governments and the media
Q13.S2	Covid-19 is a normal flu or a little more
Q13.S3	Covid-19 scared/scares me

Q13.S4	The pandemic has deteriorated the possibilities of prevention and treatment by the Health System
Q13.S5	Long-Covid is a risk that I would not like to take
Q13.S6	It is easy to avoid the virus that causes Covid-19 disease
Q13.S7	Children and young people, even if they get sick, never have problems
Q13.S8	The disease is dangerous only for elderly people and those with pathologies
Q14	Have you or a family member or houseold had Covid-19 (positive swab)?
	Yes<br>No<br>I prefer not to answer
Q15	Have you or a family member or houseold had symptoms or have you been hospitalized for Covid-19?
	Yes<br>No<br>I prefer not to answer
Q16	Has a family member or houseold died due to Covid-19?
	Yes<br>No<br>I prefer not to answer
**Data on perception of the anti-Covid vaccination**	
Q17	We now ask you to rethink the information you have received on anti-Covid vaccines: how much do you agree with the following statements? Cast a vote from 1 (disagree) to 5 (fully agree)
Q17.S1	Authorized vaccines are still experimental
Q17.S2	Many dangerous health effects of vaccines are unknown
Q17.S3	Vaccines are safe for me
Q17.S4	Vaccines are useless to contain the spread of the virus
Q17.S5	Vaccines will give rise to dangerous variants of the virus
Q17.S6	Vaccines are produced in ways that I cannot accept for religious reasons

Q17.S7	Vaccines are the best way to avoid deaths and hospitalizations
Q17.S8	mRNA vaccines (Pfizer and Moderna) are safe, adenoviral vector vaccines (AstraZeneca and J&J) are not
Q18	What are your main sources of information on vaccines? Express a vote from 1 ("Not authoritative") to 5 ("Most authoritative source"), express 0 for "Never consulted"
Q18.S1	Family Doctor or Paediatrician
Q18.S2	Doctor/s within the National Health System (Public Hygiene Service, Community Paediatrics, hospital doctors)
Q18.S3	Traditional TV and media (newspapers, magazines) or social network sites/channels connected to them
Q18.S4	Religious personalities (priests, imams, rabbis...)
Q18.S5	Social networks (Facebook groups, Twitter, Instagram, Whatsapp, Telegram…)
Q18.S6	Social dissemination profiles (university professors, researchers or popularizers such as, by way of example only: Roberta Villa, Roberto Burioni, Guido Silvestri, Antonella Viola)
Q18.S7	Friends/acquaintances
Q18.S8	Websites and/or institutional social channels (ISS, AIFA, Ministry of Health, World Health Organization, ECDC...)
Q18.S9	Counter information websites
Q18.S10	Independent doctors
Q19	Have you done or booked the anti-Covid vaccination?
	Yes<br>No<br>I prefer not to answer
Q20	Would you recommend anti-Covid vaccination to your friends/acquaintances?
	Yes<br>No<br>I prefer not to answer
**Data on perception of anti-Covid vaccination in adolescents 12-17 years old**	

Q21	We ask you to focus now on the possibility of vaccinating your children between 12 and 17 years old: how much do you agree with the following statements? Cast a vote from 1 (disagree) to 5 (fully agree)
Q21.S1	I have vaccinated/I will definitely have my children vaccinated
Q21.S2	I am not going to vaccinate my kids
Q21.S3	The vaccine has an excellent level of safety
Q21.S4	The vaccine has not been sufficiently tested
Q21.S5	I want to wait before I vaccinate my children
Q21.S6	My children are healthy, they don’t need vaccines for them
Q21.S7	I have vaccinated/will vaccinate my children because they are vulnerable due to pathological conditions
